# Aktuelle Befunde zur Koinzidenz von zerebraler Amyloidangiopathie und Alzheimer-Erkrankung

**DOI:** 10.1007/s00115-021-01213-x

**Published:** 2021-10-15

**Authors:** R. Haußmann, P. Homeyer, M. Donix, J. Linn

**Affiliations:** 1 Universitäts DemenzCentrum (UDC), Klinik und Poliklinik für Psychiatrie und Psychotherapie, Universitätsklinikum Carl Gustav Carus an der Technischen Universität Dresden, Dresden, Deutschland; 2 DZNE, Deutsches Zentrum für Neurodegenerative Erkrankungen, Dresden, Deutschland; 3 Institut und Poliklinik für diagnostische und interventionelle Neuroradiologie, Universitätsklinikum Carl Gustav Carus an der Technischen Universität Dresden, Dresden, Deutschland

**Keywords:** Zerebrale Amyloidangiopathie, Demenz vom Alzheimer-Typ, Kognition, Amyloidmetabolismus, Mikroangiopathie, Cerebral amyloid angiopathy, Alzheimer’s disease, Cognition, Amyloid metabolism, Microangiopathy

## Abstract

Die zerebrale Amyloidangiopathie (CAA) tritt trotz verschiedener Pathomechanismen häufig koinzident zur Alzheimer-Demenz auf. Sie moduliert kognitive Defizite im Rahmen der Alzheimer-Erkrankung (AD) annehmbar durch additive Effekte, auch wenn die diesbezüglichen Zusammenhänge komplex sind. Die pathophysiologische Gemeinsamkeit beider Erkrankungen besteht in einem gestörten Amyloidmetabolismus, distinkt ist jedoch die pathologische Prozessierung von Amyloidvorläuferproteinen. Die CAA mit ihren verschiedenen Subtypen ist eine pathomechanistisch heterogene Gefäßerkrankung des Gehirns. Vaskuläre und parenchymatöse Amyloidablagerungen kommen gemeinsam, aber auch isoliert und unabhängig voneinander vor. Um den spezifischen Beitrag der CAA zu kognitiven Defiziten im Rahmen der AD zu untersuchen, bedarf es daher geeigneter diagnostischer Methoden, die der Komplexität der histopathologischen bzw. bildmorphologischen Charakteristika der CAA gerecht werden, sowie differenzierender testpsychometrischer Verfahren, anhand derer der Beitrag der CAA zu kognitiven Defiziten deskriptiv erfasst und damit ätiologisch besser zuordenbar wird.

## Abstract (en)

Cerebral amyloid angiopathy (CAA) is closely related to Alzheimer’s disease (AD) despite having distinct pathomechanisms. The CAA modulates cognitive impairment within AD by synergistic effects. The pathophysiologic relations are complex and incompletely understood, possibly due to the heterogeneous nature of CAA with its different subtypes. Both diseases are characterized by a pathologic amyloid metabolism but the pathologic processing of amyloid precursor proteins is distinct. The manifestation of vascular and parenchymal amyloid deposits can either overlap or occur independently and isolated. The investigation of the specific contribution of co-occurring CAA within AD to cognitive deficits requires diagnostic methods that sufficiently identify CAA severity and complexity as well as detailed neuropsychological testing to precisely characterize the cognitive deficits and to draw conclusions regarding their etiology.

## Hintergrund

Bei der zerebralen Amyloidangiopathie (CAA) handelt es sich um eine zerebrovaskuläre Erkrankung, die durch die extrazelluläre Ablagerung von β‑Amyloid in die Gefäßwand kleiner bis mittelgroßer zerebraler und leptomeningealer Arterien, Ateriolen, Venen und Kapillaren gekennzeichnet ist [1, 2]. Im höheren Lebensalter stellt die CAA eine häufige Ursache intrazerebraler Blutungen und kognitiver Störungen dar [3]. Die Inzidenz der CAA ist, wie die Manifestation einer Demenz vom Alzheimer-Typ (AD), im hohen Maße altersabhängig [4]. Schätzungen ergeben, dass sich bei > 80-Jährigen in nur 25–40 % der Fälle keine histologischen Anzeichen einer CAA finden lassen [1].

Bei Patienten mit einer Alzheimer-Demenz ist in über 80 % der Fälle auch eine CAA nachweisbar [1]. Die Erkrankung ist nachgewiesenermaßen mit dem Vorliegen einer Alzheimer-Pathologie, dem Vorhandensein eines klinischen Alzheimer-Demenz-Syndroms (OR = 1,237; 95 %-KI 1,082–1,414) sowie mit einem beschleunigten kognitiven Abbau, insbesondere in mnestischen Domänen sowie im Bereich der Verarbeitungsgeschwindigkeit, assoziiert, tritt aber auch isoliert im höheren Lebensalter auf [1, 5].

Doch trotz der Assoziationen, die zwischen CAA und AD bestehen, handelt es sich um zwei distinkte diagnostische Entitäten [3]. Einerseits finden sich zwar in nahezu allen Fällen einer AD histopathologische Veränderungen im Sinne einer CAA und in 25 % der Fälle sogar eine weit fortgeschrittene CAA-Pathologie, andererseits erfüllen aber weniger als 50 % der CAA-Fälle die pathologischen AD-Kriterien und > 75 % der Patienten mit Alzheimer-Demenz weisen lediglich eine milde CAA-Ausprägung oder keine CAA auf [3]. Ferner ist davon auszugehen, dass > 30 % der Älteren > 70 Jahre mit CAA asymptomatisch sind [3].

Vor diesem Hintergrund gelten die genauen pathophysiologischen Zusammenhänge zwischen CAA und AD und insbesondere die exakten Pathomechanismen der Entstehung kognitiver Defizite im Rahmen der CAA mit oder ohne AD als komplex und derzeit unvollständig verstanden. Der pathophysiologische Zusammenhang besteht bekanntermaßen darin, dass beide Erkrankungen mit einem gestörten Amyloidmetabolismus einhergehen, distinkt ist jedoch die pathologische Prozessierung von Amyloidvorläuferproteinen. Diese Übersichtsarbeit beschäftigt sich daher mit der Pathophysiologie, klinischen Präsentation, Diagnostik und mit therapeutischen Ansätzen der CAA sowie mit Assoziationen der CAA zu kognitiven Defiziten im Rahmen der AD und der Frage, welchen spezifischen Beitrag eine koinzident zur AD vorliegende CAA zu kognitiven Defiziten leistet.

## Pathophysiologie

Die genaue Herkunft der im Rahmen der CAA abgelagerten β‑Amyloid-Peptide in zerebralen Gefäßen ist aktuell nicht vollständig geklärt [3]. Wahrscheinlich handelt es sich um überwiegend von Neuronen synthetisiertes β‑Amyloid, welches sich nachfolgend in Gefäßwänden ablagert [6]. Andere Hypothesen, die ein primär peripher und vor allem hepatogen synthetisiertes β‑Amyloid als Quelle vermuten, gelten hingegen als weniger wahrscheinlich [3, 7], da in der Pathogenese der CAA eine zentrale Störung der perivaskulären β‑Amyloid-Clearance angenommen wird, in deren Rahmen weniger neuronal synthetisiertes β‑Amyloid die Blut-Hirn-Schranke ins periphere Kompartiment überwindet [8]. Bei den Gefäßwandablagerungen handelt es sich u. a. um Aβ1-40- und Aβ1-42-Peptide, wobei das kürzere Aβ1-40-Peptid überwiegt [9]. Im Vergleich zu parenchymatösen Amyloidplaques weisen die vaskulären Amyloidablagerungen eine größere Aβ1-40:Aβ1-42-Ratio auf [8]. Das kürzere Aβ1-40 ist besser löslich, diffundiert leichter entlang der perivaskulären Drainagewege und akkumuliert daher vermehrt in der Gefäßwand [8]. Möglichweise stammt das kürzere und besser lösliche Aβ1-40-Peptid aus glatten Muskelzellen [8]. Die vaskulären Amyloidablagerungen werden vor allem in posterioren und insbesondere okzipitalen Hirnstrukturen gefunden, seltener in der weißen Substanz, dem Hirnstamm oder den Basalganglien [8].

Je nach Lokalisation der Amyloidablagerung wird die Unterteilung der CAA in 2 Subformen postuliert [8]. Bei der CAA‑I findet die Amyloidablagerung überwiegend in Hirnkapillaren statt und zeigte eine diffuse Verteilung im Neokortex und Hippokampus. Neuropathologische Studien zeigen Assoziationen der CAA‑I mit hippokampalen Mikroinfarkten und kognitiven Defiziten [10]. Hinsichtlich der Entwicklung kognitiver und insbesondere mnestischer Defizite außerhalb zerebraler Blutungsereignisse stellt die CAA‑I annehmbar die relevantere Subform dar, auch weil diese Assoziationen zu einer schweren Alzheimer-Pathologie aufweist. Bei der CAA-II sind die zerebralen Kapillaren hingegen ausgespart. Dieser Subtyp betrifft größere kortikale Gefäße und gilt daher als Risikofaktor für Hämorrhagien [8].

Auf genetischer Ebene sind sowohl das ApoE2- als auch das ApoE4-Allel Risikofaktoren für das Auftreten einer sporadischen CAA und auch für rezidivierende Mikroblutungen im Verlauf der Erkrankung [3, 8]. Das ApoE2-Allel, welches im Kontext der AD als protektiv gilt, ist mit dem Auftreten der nichtkapillären CAA-II vergesellschaftet und stellt daher einen Risikofaktor für Hämorrhagien dar, während das ApoE4-Allel, welches auch einen bedeutsamen genetischen Risikofaktor für die sporadische AD darstellt, das Risiko für eine schwere kortikale CAA mit hoher Amyloidlast erhöht [8] und insbesondere Assoziationen zur kapillären CAA-I-Subform und damit zur Entwicklung kognitiver Defizite aufweist [8]. Die Assoziationen zwischen dem ApoE2-Allel und intrazerebralen Blutungen wurden wiederholt gezeigt, während das ApoE4-Allel häufiger bei nichthämorrhagischen CAA-Verläufen gefunden wird [11].

Wie auch bei der Alzheimer-Erkrankung begünstigt die Beeinträchtigung der perivaskulären β‑Amyloid-Clearance bei der CAA die β‑Amyloid-Ablagerung in der Gefäßwand [8]. Diese führt konsekutiv zur Stenosierung von Blutgefäßen, die wiederum maßgebliche Ursache für die bei der CAA häufig beobachteten kortikalen Mikroinfarkte und subkortikalen Läsionen der weißen Substanz ist, welche insbesondere bei der fortgeschrittenen CAA signifikant häufiger gefunden werden als bei gesunden Älteren oder beim Vorliegen einer isolierten Alzheimer-Erkrankung [3, 12–14]. Die für die CAA charakteristischen Mikroinfarkte finden sich typischerweise im Kortexband oder in der unmittelbar darunterliegenden weißen Substanz [3]. Bei etwa 15 % der Patienten mit CAA findet man in diffusionsgewichteten Sequenzen (DWI) hyperintense Läsionen [15, 16], welche in Anbetracht der transienten Natur DWI-hyperintenser Läsionen eine große Häufigkeit subakuter, mehrheitlich klinisch stummer Infarkte im Rahmen der CAA suggerieren [3].

Neben den häufigen rezidivierenden Mikroinfarkten stellen auch zerebrale Mikroblutungen ein histopathologisches sowie bildmorphologisches Charakteristikum der CAA dar. Diese typischerweise lobären Mikroblutungen sind das Resultat einer vermehrten Gefäßfragilität und Rupturneigung [17]. Mikroblutungen im Rahmen der CAA treten, da sie ebenfalls Folge vaskulärer Amyloidablagerungen sind, ebenfalls vorzugsweise in temporalen und okzipitalen Kortexarealen auf [18]. Sie sind mit dem Fortschreiten der CAA-Pathologie und auch mit CAA-assoziierten kognitiven Einbußen assoziiert [19, 20]. Insbesondere die Anzahl der Mikroblutungen bei Erstuntersuchung ist mit dem Auftreten kognitiver Defizite im weiteren Erkrankungsverlauf vergesellschaftet [19]. Zum anderen weisen Patienten mit isoliert vorliegender AD im Vergleich zu Gesunden signifikant häufiger und vergleichsweise mehr Mikroblutungen auf [21, 22].

## Klinische Präsentation der CAA

Die bekannteste klinische Manifestation der CAA sind große, intrazerebrale Lappenblutungen, die sich mit einer akuten Schlaganfallsymptomatik präsentieren und häufig rezidivierend auftreten. Risikofaktoren für rezidivierende intrazerebrale Blutungen im Rahmen der CAA stellen dabei insbesondere die sog. superfizielle kortikale Siderose (s. unten), zurückliegende Makroblutungen, zahlreiche zerebrale Mikroblutungen (s. unten), T2-Hyperintensitäten der posterioren weißen Substanz und die Behandlung mit Thrombozytenaggregationshemmern dar [11, 23].

Neben den Makroblutungen kann die CAA auch zu sog. transienten fokal-neurologischen Episoden (TFNE) führen, d. h. zu kurzzeitigen Sensibilitätsstörungen oder motorischen Defiziten. Diese ähneln klinisch einer transienten ischämischen Attacke (TIA), sind jedoch nicht ischämisch verursacht, sondern durch akute fokale Subarachnoidalblutungen und/oder durch eine sog. kortikale superfizielle Siderose [2, 24–26]. Diese zu erkennen und von einer TIA abzugrenzen ist für das klinische Management essenziell, da die nach einer TIA indizierte, sekundärprophylaktische Therapie mit Thrombozytenaggregationshemmern hier kontraproduktiv wäre, da sie das Blutungsrisiko erhöht [26].

Zusätzlich zu diesen akuten Präsentationen führt die CAA auch zu kognitiven Einschränkungen mit demenzieller Entwicklung. Insbesondere bei symptomatischen Hämorrhagien im Bereich des Frontallappens sind aber auch atypische kognitive Manifestationen in Form dysexekutiver Syndrome möglich [25]. Trotz der großen Häufigkeit der CAA im höheren Lebensalter kann die klinische Diagnosestellung herausfordernd sein [24], denn die klinischen Zeichen und die bildmorphologischen Charakteristika sind weder spezifisch noch sensitiv [24].

## Diagnostik

Für die Diagnosestellung einer CAA wurden klinische und radiologische Kriterien validiert (Boston-Kriterien; [27]), die erstmals 1985 publiziert und 2010 modifiziert worden sind [28, 29]. Für die definitive Diagnosestellung einer CAA gemäß dieser Kriterien bedarf es einer umfassenden histopathologischen Untersuchung post mortem. Grundlegende Erkenntnisse zur Histopathologie der CAA stammen dabei aus pathologischen Untersuchungen nach Hämatomevakuationen, Hirnbiopsien und Obduktionen in den 1980er-Jahren [17].

Ohne invasive Gewebediagnostik kann in vivo maximal die Diagnose einer wahrscheinlichen CAA gestellt werden. Gemäß der modifizierten Boston-Kriterien erfordert diese den bildgebenden Nachweis mehrerer lobär – also oberflächlich – lokalisierter Blutungen oder mindestens einer solchen Blutung und einer sog. kortikalen superfiziellen Siderose (s. unten). Obwohl weder die initiale noch die modifizierte Version der Boston-Kriterien eine Angabe zur Größe der hier zu berücksichtigenden Blutungen machen, werden heute in der praktischen Anwendung die sog. zerebralen Mikroblutungen in diesem Zusammenhang meist auch als „Blutung“ gezählt [30]. Zerebrale Mikroblutungen (CMB) sind kleine parenchymale Hämosiderinablagerungen, die nur auf hämosiderinsensitiven, T2*-gewichteten Gradientenecho-MRT-Sequenzen erkennbar sind [31]. Bei der CAA sind diese CMB typischerweise oberflächlich, also kortikal oder unmittelbar subkortikal an der Mark-Rinden-Grenze lokalisiert, während sie bei der hypertensiven Arteriopathie, einer weiteren häufigen zerebralen Mikroangiopathie, bevorzugt in der tiefen grauen Substanz und dem Hirnstamm lokalisiert sind. Diese Regionen sind bei der CAA dagegen charakteristischerweise ausgespart [32].

Die als eigenständiger Befund in den Boston-Kriterien aufgeführte kortikale superfizielle Siderose (cSS) ist definiert als lineare Hämosiderinablagerungen in den Sulci und/oder den oberflächlichen kortikalen Schichten der Großhirnhemisphären und ist ebenfalls nur mittels T2*-gewichteter MR-Sequenzen detektierbar. Sie stellt einen sehr spezifischen MRT-Marker der CAA dar, der bei anderen zerebralen Mikroangiopathien, wie z. B. der CADASIL-Erkrankung, nicht gefunden wird [33]. Ist die cSS auf weniger als vier kortikale Furchen beschränkt, spricht man von einer fokalen cSS, sind vier oder mehr Sulci betroffen, liegt eine disseminierte cSS vor. Insbesondere letztere Ausprägung ist mit kognitiven Beeinträchtigungen assoziiert [34–36]. Das Vorliegen einer cSS erhöht darüber hinaus das Risiko für rezidivierende intrakranielle Blutungen deutlich, insbesondere in Lokalisationen der vorbestehenden cSS [30, 37].

Neben den hämorrhagischen Veränderungen, werden bei der CAA auch nichthämorrhagische Parenchymveränderungen gefunden: posterior gelegene T2-Hyperintensitäten der weißen Substanz [27], kortikale Mikroinfarkte und erweiterte perivaskuläre Räume im Centrum semiovale.

Um die Gesamtlast der CAA-assoziierten intrazerebralen Pathologien insbesondere im Rahmen von Studien abzubilden, wurde der sog. Total-MRI-vessel-disease-Score vorgeschlagen, der zerebrale Mikroblutungen, die kortikale superfizielle Siderose, erweiterte perivaskuläre Räume im Centrum semiovale und T2-Hyperintensitäten der weißen Substanz berücksichtigt und graduiert und deutliche Assoziationen zur histopathologischen CAA-Schwere, zu symptomatischen intrazerebralen Blutungen und DWI-positiven Läsionen in der posterior gelegenen weißen Substanz aufweist [38].

Weitere Biomarker, deren diagnostischer Wert Gegenstand verschiedener Untersuchungen war, sind Liquorparameter und der intrazerebrale Amyloidnachweis in der Positronenemissionstomographie (Amyloid-PET). Daten aus Liquoruntersuchungen zeigen erniedrigte Aβ1-40- und Aβ1-42-Proteinkonzentrationen im Liquor bei Patienten mit CAA im Vergleich zu gesunden Kontrollen, insbesondere bei Patienten mit superfizieller kortikaler Siderose und oberflächlichen Subarachnoidalblutungen [39–41].

Mittels Amyloid-PET kann die Amyloidablagerung quantifiziert und auch lokalisiert werden [42, 43]. Im Vergleich zu Gesunden misst man bei der CAA mehr, im Vergleich zur AD weniger Amyloidablagerung in der Amyloid-PET [3]. Eine Besonderheit der CAA im Vergleich zur AD ist dabei die vermehrte okzipitale Amyloidablagerung. So wurde bei der CAA eine signifikant größere Okzipital:Global-Ratio hinsichtlich der Amyloidablagerung gemessen [42]. Aktuelle PET-Daten zeigen außerdem, dass insbesondere frontale und parietale Hyperintensitäten der weißen Substanz Assoziationen zu Amyloidablagerungen zeigen, während derartige Beziehungen zu Tau-Protein-Ablagerungen nicht nachgewiesen wurden [44]. Insbesondere die amyloidassoziierten Marklagerläsionen korrelieren wiederum stark mit dem Vorliegen von CMB, was einen Beitrag der CAA zu den Assoziationen zwischen Amyloidablagerungen und Marklagerveränderungen suggeriert [44].

Doch Liquorparameter und das Amyloid-PET haben in der Diagnostik der CAA bislang keinen klinischen Stellenwert, sondern sind bislang lediglich Gegenstand wissenschaftlicher Analysen.

## Assoziationen der CAA zur Demenz vom Alzheimer-Typ und zu kognitiven Defiziten

Der grundsätzliche Beitrag einer vaskulären Pathologie zur allgemeinen Entwicklung kognitiver Einbußen findet zunehmende Berücksichtigung in der aktuellen Literatur [45]. Neuropathologische Studien, aktuelle Befunde aus der Bildgebung sowie Liquordaten lassen vermuten, das mikroangiopathische Veränderungen zu 50 % aller Demenzen, inklusive der Alzheimer-Demenz, beitragen [45]. In diesem Zusammenhang werden insbesondere gefäßschädigende Effekte von Aβ- und Tau-Protein diskutiert [46, 47]. Aktuelle Daten zeigen, dass insbesondere der hippokampale Niedergang der Blut-Hirn-Schranke und eine begleitende kapilläre Pathologie ein früher, von einer Amyloid- sowie Tau-Pathologie unabhängiger Biomarker in der Entwicklung kognitiver Einbußen ist [45].

Insbesondere die Interaktion zwischen CAA und AD hinsichtlich der Entwicklung kognitiver Defizite ist komplex. Gemeinsamkeiten liegen in der zentralen pathophysiologischen Bedeutung der Aβ-Produktion und -Clearance, während Unterschiede unter anderem im Ort der Amyloidablagerung (vaskulär vs. parenchymatös) und in der Häufigkeit verschiedener Aβ-Peptid-Subspezies liegen [8]. Die AD und die CAA weisen grundlegend verschiedene Pathomechanismen auf [8]. Während AD-assoziierte neurotoxische Effekte wesentlich über den β‑Amyloid-getriggerten Verlust von Synapsen und Neuronen vermittelt werden, scheint der neurotoxische Mechanismus der CAA im Wesentlichen Folge einer vaskulären Pathologie mit Verlust der Gefäßintegrität, Hämorrhagien und Ischämien zu sein [8].

Neuropathologische Erkenntnisse lassen vermuten, dass vaskuläre und parenchymatöse Amyloidablagerungen relativ unabhängig voneinander auftreten, aber auch überlappend vorkommen [3].

Nach aktuellem Verständnis leistet eine bei der AD komorbid vorliegende CAA einen unabhängigen Beitrag zur kognitiven Beeinträchtigung in Form additiver Effekte durch eine zusätzlichen Beeinträchtigung der perivaskulären β‑Amyloid-Clearance und additiv hinzutretende zerebrovaskuläre Läsionen [3, 8, 48, 49]. Insbesondere die Beeinträchtigung der perivaskulären β‑Amyloid-Drainage trägt zum rascheren Fortschreiten einer AD-Pathologie bei [50]. Bei der CAA treten jedoch auch in Abwesenheit einer ausgeprägten Alzheimer-Pathologie kognitive Defizite auf [51].

In Übereinstimmung mit der Hypothese zu additiven dyskognitiven Effekten findet man bei Patienten mit Alzheimer-Demenz und komorbider CAA ausgeprägtere kognitive Defizite als bei Patienten mit isoliert vorliegender AD [49, 52]. Zudem korrelieren CAA- und AD-Schwere miteinander [53].

Multivariate Analysen, die für das Vorliegen einer AD und für zerebrale Infarkte kontrollieren, zeigen, dass insbesondere Defizite in den Domänen Verarbeitungsgeschwindigkeit und episodisches Gedächtnis mit einer mittelschwer bis schwergradig ausgeprägten CAA assoziiert sind [53]. Kognitive Defizite im Rahmen der CAA sind zusammenfassend am ehesten multifaktoriell bedingt. Neben der pathologischen, multilokulären Amyloidablagerung spielt annehmbar auch das Vorhandensein der unterschiedlichen CAA-Subtypen eine Rolle, von denen insbesondere die kapilläre CAA‑I mit hippokampalen Mikroinfarkten assoziiert ist, was als pathomechanistisches Korrelat der episodischen Neugedächtnisstörung diskutiert werden kann [10], während die zerebrovaskuläre Pathologie die herabgesetzte Verarbeitungsgeschwindigkeit erklärt.

Bezüglich einer möglichen ischämischen Genese kognitiver Defizite bei der CAA stellt die Menge an β‑Amyloid-beladenen und konsekutiv stenosierten Gefäßen im Rahmen der CAA einen vom Vorliegen einer parenchymatösen Amyloidpathologie unabhängigen Prädiktor für die Entwicklung eines demenziellen Syndroms dar [54]. Ferner ist die Schädigung der weißen Substanz im Rahmen der CAA unabhängig von den Effekten durch Hirnblutungen mit kognitiven Defiziten assoziiert [12, 55]. Mehrere Studien konnten darüber hinaus zeigen, dass auch das Vorliegen einer CAA-assoziierten kortikalen superfiziellen Siderose – insbesondere in ihrer disseminierten Form – mit kognitiven Defiziten assoziiert ist [34–36].

Will man die Effekte der CAA auf das kognitive Funktionsniveau von Patienten mit AD untersuchen, müssen diese von dyskognitiven Effekten einer zerebralen Mikroangiopathie differenziert werden. Denn grundlegend bahnen auch mikroangiopathische Läsionen die Manifestation und Schwere klinischer Symptome im Rahmen der AD. Vorrangig lakunäre Infarkte im Bereich der Basalganglien, des Thalamus und der tiefer gelegenen weißen Substanz sind beim Vorliegen einer Alzheimer-Pathologie mit einer hohen Demenzprävalenz assoziiert, während lakunäre Infarkte bei Patienten ohne begleitende Alzheimer-Pathologie nur schwache Assoziationen mit einem kognitivem Abbau aufweisen [49]. Insbesondere in frühen Stadien der AD beeinflussen zerebrovaskuläre Läsionen das kognitive Funktionsniveau stärker als in Fällen fortgeschrittener Veränderungen [52]. Bei der Untersuchung spezifischer kognitiver Defizite im Rahmen der CAA und deren Ätiologie müssen diese Aspekte Berücksichtigung finden.

## Therapeutische Ansätze

Bislang existieren keine zugelassenen oder evidenzbasierten medikamentösen Behandlungsoptionen zur spezifischen Therapie der CAA. Vor diesem Hintergrund kommt insbesondere der Prävention von Gefäßveränderungen eine hohe Bedeutung zu. Da eine Gefäßintegrität von zentraler Bedeutung bei der perivaskulären Aβ-Clearance ist, ist der Erhalt der vaskulären Struktur und Funktion durch Modifikation der typischen Gefäßrisikofaktoren von zentraler Bedeutung bei der Prävention und Modulation einer CAA-Manifestation [8]. Insbesondere die Blutdrucksenkung kann das Risiko für intrazerebrale Blutungen bei Patienten mit CAA deutlich reduzieren [56].

Hinsichtlich der Therapie mit Antikoagulanzien sollte insbesondere eine Behandlung mit Falithrom, wenn möglich, vermieden werden. Neue orale Antikoagulanzien (NOAK) sind eine Therapieoption für Patienten mit Vorhofflimmern und einem hohen Risiko für kardioembolische zerebrale Ischämien. Bei Patienten mit CAA und Vorhofflimmern und daraus resultierendem hohem Schlaganfallrisiko stellt jedoch auch der Verschluss des linken Vorhofohres eine mögliche Therapieoption dar, die in Einzelfällen erwogen werden sollte [57]. Die Indikation für eine Thrombozytenaggregationshemmung muss bei Patienten mit CAA in Anbetracht des erhöhten Blutungsrisikos kritisch geprüft werden [23], wenngleich das Blutungsrisiko unter Thrombozytenaggregationshemmung geringer ist als unter Antikoagulanzien.

## Zusammenfassung und Ausblick

Klinische sowie bildmorphologische Charakteristika der CAA sind komplex. Die CAA tritt häufig koinzident zu einer bestehenden Alzheimer-Erkrankung auf. Insbesondere der pathomechanistische Beitrag der CAA zu kognitiven Defiziten im Rahmen der AD ist komplex und aktuell unvollständig verstanden. Hinsichtlich der Pathomechanismen beider Erkrankungen handelt es sich bei der CAA und der AD jedoch um distinkte diagnostische Entitäten mit annehmbar additiven Effekten hinsichtlich kognitiver Abbauprozesse, wobei aber insbesondere die fortgeschrittene CAA einen von der AD und zerebralen Infarkten unabhängigen Beitrag zur Entstehung kognitiver Defizite zu leisten scheint. Eine weitere Ursache für die Komplexität der klinischen und bildmorphologischen Zeichen der CAA ist das Vorhandensein verschiedener CAA-Subtypen mit unterschiedlich lokalisierten Amyloidablagerungen und verschiedenartigen Assoziationen zur Entwicklung kognitiver Defizite und Hämorrhagien. Charakteristisch und in der Abgrenzung zu anderen vaskulären zerebralen Erkrankungen des Gehirns relevant ist das typischerweise posteriore, temporookzipitale Verteilungsmuster der CAA, was insbesondere in der Abgrenzung zur hypertensiv bedingten zerebralen Mikroangiopathie von differenzialdiagnostischer Bedeutung ist. Das Wissen um die Diagnose einer CAA ist insbesondere aufgrund des signifikant erhöhten Risikos für Makroblutungen unter der Therapie mit Antikoagulanzien von großer klinischer Bedeutung. Ferner stellen oberflächliche Subarachnoidalblutungen im Rahmen einer kortikalen superfiziellen Siderose aufgrund ihrer klinischen Präsentation in Form transienter fokal-neurologischer Defizite eine wichtige Differenzialdiagnose transienter ischämischer Episoden dar.

Vor Einführung hämosiderinsensitiver, T2*-gewichteter Gradientenecho-MRT-Sequenzen war insbesondere die fehlende Möglichkeit, die Schwere der CAA standardisiert und vollständig zu erfassen, Hindernis bei der Erforschung CAA-spezifischer Effekte auf die Kognition [51]. Bis heute ist beispielsweise relativ wenig darüber bekannt, welche kognitiven Domänen charakteristischerweise durch das Vorliegen einer CAA beeinträchtigt werden. Die wenigen Untersuchungen diesbezüglich weisen auf eher unspezifische Veränderungen der Verarbeitungsgeschwindigkeit sowie dem episodischen Gedächtnis hin. Aufgrund des schwerpunktmäßig kortikoposterioren Befallsmusters der CAA wären spezifischere Defizite, vorrangig vom kortikalen Typ und beispielsweise in sprachlichen Domänen, Praxie und Visuokonstruktion, zu erwarten. Zur Prüfung dieser Hypothese geeignete Untersuchungen bedürften dem parallelen Erfassen der bildmorphologischen CAA-Schwere und -Komplexität mit geeigneten Score-Systemen, einer Kontrolle für das Vorliegen einer nicht-CAA-assoziierten zerebralen Mikroangiopathie sowie der Erhebung standardisierter, differenzierend neuropsychologischer Testbefunde. Der Vergleich testpsychometrischer Befunde in relevanten diagnostischen Entitäten kognitiver Störungen, wie leichten kognitiven Störungen, Alzheimer- oder gemischten Demenzen mit und ohne CAA könnte diesbezüglich Daten liefern, die Rückschlüsse auf den spezifischen Beitrag der CAA zu kognitiven Defiziten zulassen.
